# Supplementary material for: Real-Time Viability Assessment of Ex Vivo Mouse Kidneys for Transplant Applications Using Dynamic Optical Coherence Tomography
Source: bioRxiv. 2025 Sep 4:2025.08.30.673280. Preprint. [Version 1] doi: 10.1101/2025.08.30.673280 (PMC12424824; doi:10.1101/2025.08.30.673280)
Supplement: Supplement 1 [file media-1.docx]

Supplementary Figures


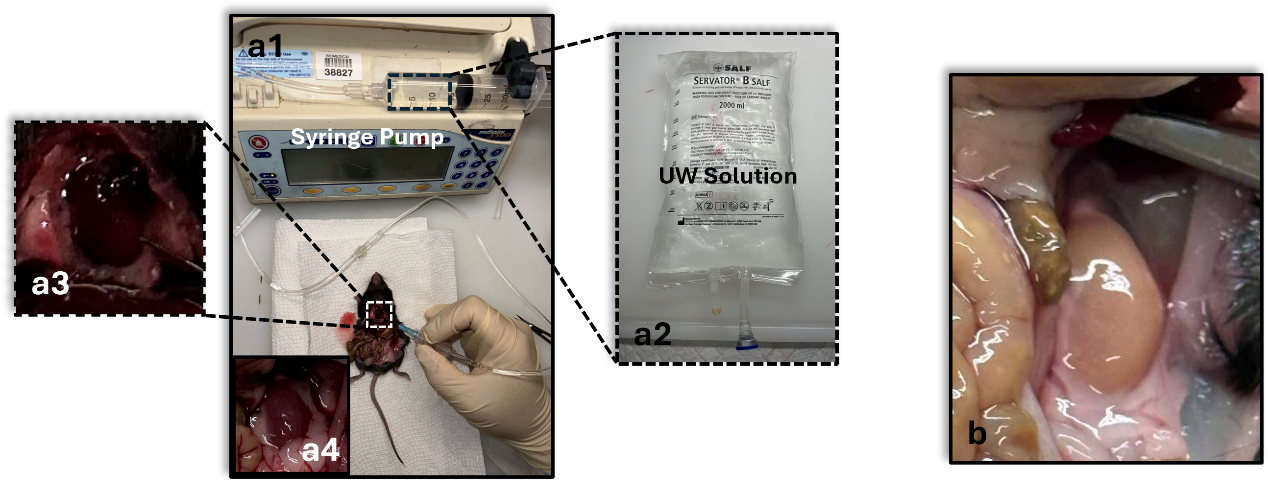


Figure S1. Experimental setup used for kidney flushing with UW solution. (a1) Experimental setup. (a2) UW solution. (a3) Insert the needle into the left ventricle. (a4) A kidney before flushing. (b) A kidney after flushing.


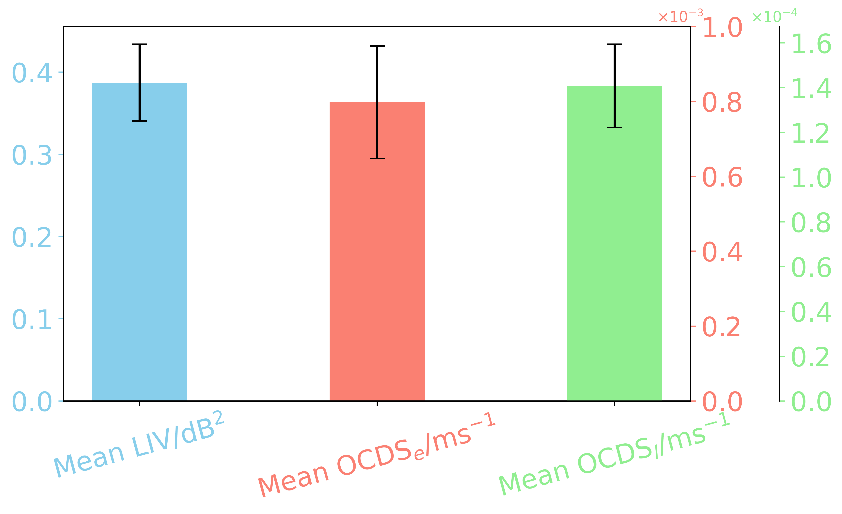


Figure S2. Mean LIV, OCDS*_e_* and OCDS*_l_* values of formalin-preserved mouse kidneys The overall mean values across all mice were 0.387, 7.96×10⁻⁴, and 1.41×10⁻⁴, respectively.
